# Suppressive Effect of Shiitake Extract on Plasma Ethanol Elevation

**DOI:** 10.3390/nu12092647

**Published:** 2020-08-31

**Authors:** Harumi Uto-Kondo, Ayaka Sakurai, Kazuki Ogawa, Yusuke Yamaguchi, Takeshi Saito, Hitomi Kumagai

**Affiliations:** 1 Department of Bioscience in Daily Life, Nihon University, 1866 Kameino, Fujisawa-shi 252-0880, Japan; kondou.harumi@nihon-u.ac.jp; 2 Department of Chemistry and Life Science, Nihon University, 1866 Kameino, Fujisawa-shi 252-0880, Japan; ayapico19940520@yahoo.co.jp (A.S.); brka19008@g.nihon-u.ac.jp (K.O.); yamaguchi.yusuke@nihon-u.ac.jp (Y.Y.); 3 ACERA Co., Ltd., 156 Nishitkahashi-machi, Kofu-shi 400-0826, Japan; t-saito@acera-jp.com

**Keywords:** shiitake flavor precursor, ethanol absorption, ethanol metabolism, blood ethanol concentration, lentinic acid

## Abstract

Alcohol is usually consumed with meals, but chronic consumption is a leading cause of alcoholic liver diseases. We investigated if shiitake extracts with a high lentinic acid content (Shiitake-H) and without lentinic acid (Shiitake-N) could suppress the elevation in plasma ethanol concentrations by accelerating ethanol metabolism and preventing ethanol absorption from the gut. Shiitake-H and Shiitake-N suppressed the elevation in concentrations of ethanol and acetaldehyde in plasma, and promoted the activities of alcohol dehydrogenase (ADH) and aldehyde dehydrogenase (ALDH) in the liver. However, these effects of Shiitake-H were more prominent than those of Shiitake-N. Furthermore, Shitake-H promoted ADH and ALDH activities in the stomach. We also examined the change in plasma ethanol concentration by injecting Shiitake-H or Shiitake-N into the ligated loop of the stomach or jejunum together with an ethanol solution. Shiitake-H suppressed the absorption of ethanol from the stomach and jejunum. In conclusion, Shiitake-H accelerates ethanol metabolism in the stomach and liver and inhibits ethanol absorption in the stomach and jejunum indicating that lentinic acid is a functional component in shiitake.

## 1. Introduction

The moderate intake of alcohol stimulates our appetite and has favorable effects on our health. However, excessive intake sometimes leads to various diseases. Moreover, chronic alcohol consumption is a main cause of alcoholic liver diseases (ALD), such as hepatic steatosis, steatohepatitis, fibrosis, and cirrhosis [1], which relates to the increase in oxidative stress, activity of the innate immune system, and concentrations of pro-inflammatory cytokines and gut-derived lipopolysaccharides [2,3,4]. Ethanol is converted to acetaldehyde, its primary metabolite, by alcohol dehydrogenase (ADH), and further to acetic acid by acetaldehyde dehydrogenase (ALDH). As acetaldehyde is largely responsible for alcohol-related symptoms in addition to mutagenic and carcinogenic lesions, its rapid metabolism to acetic acid is important to reduce the risk of health damage. Approximately half of Asian people are susceptible to acute alcohol intoxication due to a lack of ALDH2 [5], one of the enzymes for metabolizing acetaldehyde to acetic acid.

Shiitake (*Lentinula edodes*) is one of the most popular edible mushrooms and widely used in Japanese and Chinese dishes. Since shiitake is rich in protein, polysaccharides, dietary fiber, minerals, and vitamins, its consumption benefits human health [6]. Moreover, shiitake is known to have antioxidant [7], anti-inflammatory [8,9], antihypercholesterolemic [10], and antithrombotic [11] effects. Another feature of shiitake is its unique flavor that has been appreciated since the ancient times. The shiitake flavor components are volatile cyclic sulfur compounds including 1,2,3,5,6-pentathiepane (lenthionine), 1,2,4,6-tetrathiepane, and 1,2,3,4,5,6-hexathiepane [12]. Among them, lenthionine is the major component and characterizes shiitake flavor. These volatile cyclic sulfur compounds are formed from lentinic acid, a flavor precursor, by a two-step enzymatic reaction [13]. First, lentinic acid is converted to desglutamyl lentinic acid by γ-glutamyl transpeptidase, and then to sulfenic acid by cysteine sulfoxide (C-S) lyase. Two molecules of sulfenic acid produce thiosulfinate, which leads to the formation of cyclic sulfur compounds. The flavor formation of shiitake is similar to that of garlic. *S*-Allyl-L-cysteine sulfoxide (ACSO) in garlic is converted to sulfenic acid by C-S lyase and further reaction produces volatile sulfides such as diallyl disulfide and diallyl trisulfide. In addition, both lentinic acid and ACSO have sulfoxide, the amino and carboxyl groups in their structure.

Previously, we have shown that ACSO suppresses elevation in the blood ethanol concentration by accelerating ethanol metabolism and preventing ethanol absorption from the gut [14]. Other workers reported that some isothiocyanates enhance ALDH activity, though there is no evidence to suppress ethanol concentration in the blood. Sulforaphane, an isothiocyanate in broccoli, decreases blood acetaldehyde concentration and increase ALDH activity in the liver [15]. Similarly, 6-(methylsulfinyl)hexyl isothiocyanate (6-MSITC) in wasabi is reported to increase ALDH activity and induce mitochondrial ALDH2 in HepG2 cells [16]. As ACSO in garlic and isothiocyanates in Brassicaceae vegetables are sulfur-containing compounds, lentinic acid that has four sulfur atoms in its structure may have similar functions to them.

Therefore, the present study was undertaken to investigate whether shiitake extracts with high lentinic acid (Shiitake-H) and without lentinic acid (Shiitake-N) suppress the elevation in plasma ethanol concentration by accelerating ethanol metabolism and suppressing ethanol absorption from the gut. The acceleration of ethanol metabolism was evaluated by measuring the activities of ADH and ALDH in the liver and stomach after the oral administration of shiitake extract together with an ethanol solution, while the suppression of ethanol absorption was evaluated by injecting shiitake extract into the ligated loop of the stomach or jejunum together with an ethanol solution and measuring plasma ethanol concentration.

## 2. Materials and Methods

### 2.1. Materials

Ethanol (99.5%) was purchased from Kanto Chemical Co., Inc. (Tokyo, Japan). All chemicals in this study were analytical grade.

### 2.2. Preparation of Shiitake-H and Shiitake-N

Shiitake-H was prepared from 100 g fresh raw shiitake incubated in 400 mL of 100% methanol at 4 °C for 5 h to inactivate C-S lyase. Then, the shiitake was homogenized with the methanol adding a 1/3 volume of water, and the homogenate was centrifuged at 15,000× *g* for 5 min at room temperature to separate the supernatant (A). Furthermore, the shiitake residue was homogenized with methanol and a 1/3 volume of water again, and the homogenates were centrifuged at 15,000× *g* for 5 min at room temperature to separate the supernatant (B). The mixture of supernatant (A) and (B) was evaporated and concentrated to Brix 60 under vacuum.

On the other hand, Shiitake-N was prepared from 100 g fresh raw shiitake homogenized with 1500 mL water, and incubated at 40 °C for 20 min to allow the reaction by γ-glutamyl transpeptidase and C-S lyase to take place. Then, the homogenate was centrifuged at 15,000× *g* for 5 min at room temperature. The supernatant was incubated at 75 °C for 10 min and 1 volume of ethanol was added to precipitate polysaccharide. Then, it was centrifuged at 15,000× *g* for 5 min at room temperature to separate the supernatant (C). On the other hand, 1 volume of ethanol was added to the shiitake residue, followed by centrifugation at 15,000× *g* for 5 min at room temperature to separate the supernatant (D). The mixture of supernatant (C) and (D) was evaporated and concentrated to Brix 60 under vacuum. The obtained Shiitake-H and Shiitake-N were stored at −80 °C until use.

### 2.3. Quantitative Analyses of Lentinic Acid

Standard Fmoc-lentinic acid was prepared from Shiitake-H according to the method described by Hiraide et al. [17] with modifications. First, lentinic acid in Shiitake-H was fractionated with cation-exchange chromatography (IR-120B). Then, the fraction containing lentinic acid was evaporated, and lentinic acid was derivatized with 9-fluorenylmethyl chloroformate (Fmoc-Cl) in 0.4 M boric acid buffer (pH 7.4). Fmoc-lentinic acid was further purified with HPLC (LC-20AD and SPD-6A, Shimadzu, Tokyo, Japan) using an Inertsil ODS-4 5 µm 250 × 10 mm id (GL Sciences, Tokyo, Japan) as a column. The flow rate of the mobile phase was 5 mL/min. The percentage of mobile phase A containing 0.1% formic acid was 95% at the beginning and linearly reduced to 0% for 60 min by increasing the ratio of mobile phase B containing 0.1% acetonitrile. The detector wavelength was set at 266 nm. The structure and molecular weight of Fmoc-lentinic acid were confirmed by ^1^H NMR and LC-MS.

The lentinic-acid content in Shiitake-H and Shiitake-N was quantified by HPLC (Waters e2695, Waters Ltd., Milford, MA, USA) with a fluorescence Detector (Waters 2475) after derivatization with Fmoc. First, Shiitake-H and Shiitake-N were dissolved in 0.4 M boric acid buffer (pH 7.4) to be 1 mg/mL. Then, the same volume of 3 mM Fmoc-Cl was added to the solution and kept at room temperature for 10 min. The mixture was washed with pentane several times, and the aqueous phase was injected to HPLC after filtration. The components were separated with an Inertsil ODS-4 column (250 × 4.6 mm id) (GL Sciences), using a buffered solution consisting of formic acid: acetonitrile (v:v = 1:999) at a flow rate of 0.7 mL/min. The detector was set at 263 and 313 nm as excitation and emission wavelength, respectively. Fmoc-lentinic acid content was calculated based on the standard curve. The corresponding equation was as follows: Y = 89180X + 19088, *R*^2^ = 0.99997, X: Lentinic acid concentration (µg/mL), Y: Peak area (µV·s).

### 2.4. Animals

Male 7 week old Sprague-Dawley (SD) rats were purchased from Japan SLC, Inc. (Shizuoka, Japan) and randomly assigned to 3 groups: control group, Shiitake-H group, and Shiitake-N group (*n* = 4–6 in each group) in all experiments. All rats were housed in plastic cages and fed a solid feed (CE-2) for 5–8 days prior to the commencement of experiments under a controlled temperature (22  ±  1 °C) and humidity (55%  ±  15%) conditions, and a 12 h light/dark cycle (light period 7:00–19:00). All rats were given ad libitum access to water. When dissected, the rats were euthanized under isoflurane anesthesia. The animal experiments were performed in accordance with the Guidelines for Animal Experiments of the College of Bioresource Sciences, Nihon University (approval number: AP17B052 and AP18BRS102-1).

### 2.5. Ethanol, Acetaldehyde, and Acetate Concentrations in Plasma

**Figure 1:**
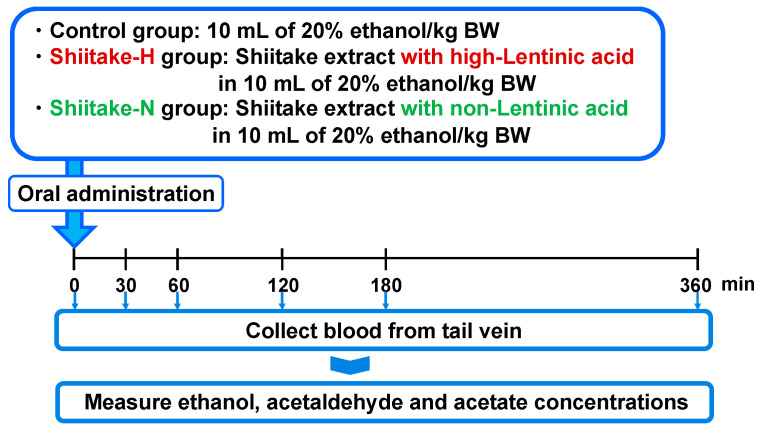
Experimental design to evaluate the effect of shiitake extracts on ethanol, acetaldehyde, and acetate concentrations in plasma. BW: body weight.

The effect of shiitake extracts on ethanol, acetaldehyde, and acetate concentrations in plasma was evaluated after orally administrating them together with 20% ethanol (Figure 1). After fasting overnight, male 7 week old SD rats were orally administered 10 mL of 20% ethanol/kg body weight (control group; body weight 190.8 ± 6.1 g), Shiitake-H containing 300 µmol lentinic acid in 10 mL of 20% ethanol/kg body weight (Shiitake-H group; body weight 184.3 ± 8.1 g), or Shiitake-N of the same amount as Shiitake-H in 10 mL of 20% ethanol/kg body weight (Shiitake-N group; body weight 183.2 ± 8.3 g). Rats in the control group were given sterile water instead of Shiitake-H and Shiitake-N solution. Before and 30, 60, 120, 180, 360 min after administration, 70 µL blood was collected by nicking the tail vein, and used to determine plasma ethanol, acetaldehyde, and acetate concentrations.

### 2.6. Ethanol Absorption from the Gut

**Figure 2:**
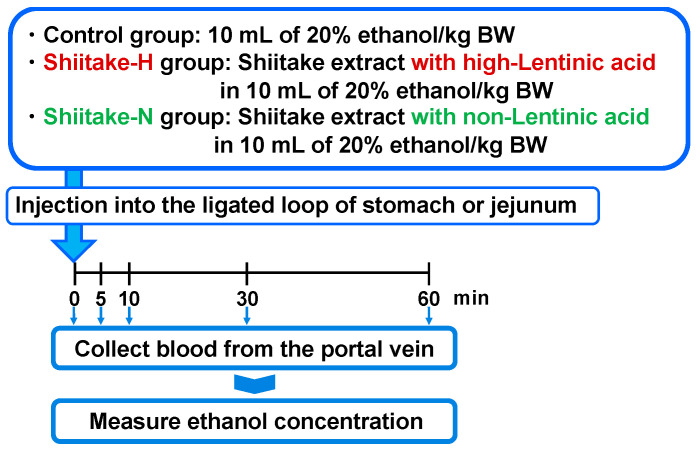
Experimental design to evaluate the effect of shiitake extracts on ethanol absorption from the gut.

In order to clarify the mechanism of actions, the suppressive effect of shiitake extracts on ethanol absorption from the gut was evaluated by injecting them together with 20% ethanol into the ligated loop of the stomach and jejunum as previously described [14,18] (Figure 2). After fasting overnight, the abdominal cavity was opened, and the upper and lower regions of the stomach or jejunum were ligated twice with 5-0 surgical silk. Then, 10 mL of 20% ethanol/kg body weight (control group; body weight 202.4 ± 8.7 g), Shiitake-H containing 300 µmol lentinic acid in 10 mL of 20% ethanol/kg body weight (Shiitake-H group; body weight 201.5 ± 10.1 g), or Shiitake-N of the same amount as Shiitake-H in 10 mL of 20% ethanol/kg body weight (Shiitake-N group; body weight 198.0 ± 7.5 g) was injected into the ligated loop of the stomach and jejunum. Before and 5, 10, 30, and 60 min after injection, 70 µL blood was collected from the portal vein and used to determine plasma ethanol concentration.

### 2.7. ADH and ALDH Activities in the Liver and Stomach

**Figure 3:**
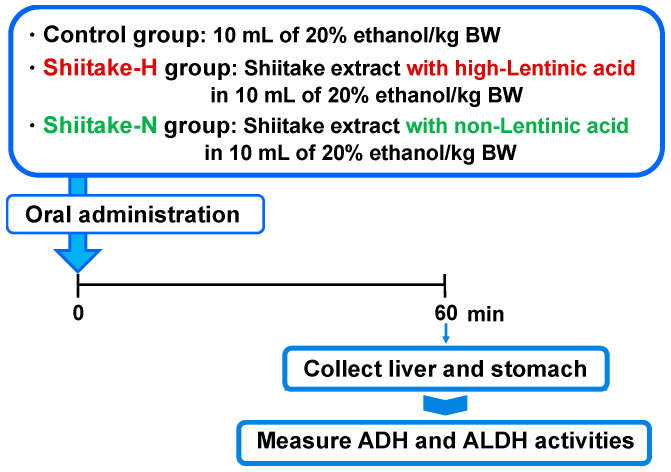
Experimental design to evaluate the effect of shiitake extracts on alcohol dehydrogenase (ADH) and aldehyde dehydrogenase (ALDH) activities.

As another possible mechanism of action, the effect of shiitake extracts on the activities of ADH and ALDH in the liver and stomach was evaluated (Figure 3). After fasting overnight, male 7 week old SD rats were orally administered by gavage 10 mL of 20% ethanol/kg body weight (control group; body weight 199.4 ± 4.3 g), Shiitake-H containing 300 µmol lentinic acid in 10 mL of 20% ethanol/kg body weight (Shiitake-H group; body weight 202.3 ± 5.7 g), or Shiitake-N of the same amount as Shiitake-H in 10 mL of 20% ethanol/kg body weight (Shiitake-N group; body weight 202.1 ± 9.3 g). After 60 min, rats were euthanized under isoflurane anesthesia, and their liver and stomach were harvested. The collected liver and stomach were immediately homogenized for the measurement of ADH and ALDH activities.

### 2.8. Residual Ethanol and Acetaldehyde Concentrations in the Stomach

**Figure 4:**
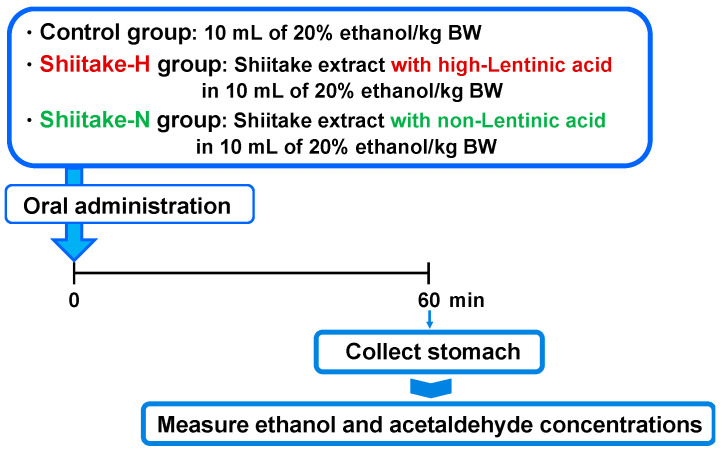
Experimental design to evaluate the effect of shiitake extracts on residual ethanol and acetaldehyde concentrations in the stomach.

To confirm the prevention of ethanol absorption from the gut by shiitake extracts, the residual ethanol and acetaldehyde concentrations in the stomach were evaluated (Figure 4). After fasting overnight, rats were orally administered by gavage 10 mL of 20% ethanol/kg body weight (control group; body weight 199.4 ± 4.3 g), Shiitake-H containing 300 µmol lentinic acid in 10 mL of 20% ethanol/kg body weight (Shiitake-H group; body weight 202.3 ± 5.7 g), or Shiitake-N in 10 mL of 20% ethanol/kg body weight (Shiitake-N group; body weight 202.1 ± 9.3 g). After 60 min, rats were euthanized under isoflurane anesthesia, and their stomach was harvested. The collected stomach was centrifuged at 10,000× *g* for 20 min at 25 °C, and the supernatant fraction was used for the measurement of ethanol and acetaldehyde concentrations.

### 2.9. Determinations of Ethanol, Acetaldehyde, and Acetate Concentrations in Plasma and Stomach

Whole blood samples collected into heparinized capillary tubes were immediately mixed with 1 M perchloric acid and kept on ice. After 10 min, the tubes were centrifuged at 10,000× *g* for 10 min at 4 °C, and the pH of supernatant fraction was adjusted to 7.5–8.0 by adding 0.7 M K_3_PO_4_. After incubation for 10 min on ice, the deproteinized plasma was isolated by centrifugation at 2300× *g* for 10 min at 4 °C. The supernatant fraction was stored at −80 °C for analysis of acetate.

Internal contents in the stomach adjusted to pH 7.6 by 6 N NaOH and 6 N HCl were centrifuged at 10,000× *g* and 25 °C for 20 min. Then, 1 mL of the supernatant fraction diluted by 99 mL sterile water was used for the measurement. Concentrations of ethanol and acetaldehyde in the plasma and stomach were determined immediately using F-kit Ethanol and F-kit Acetaldehyde (Roche Diagnostics K.K, Tokyo, Japan), respectively. The quantitative analysis of acetate was conducted by the same method as previously described [14]. 

### 2.10. Determinations of ADH and ALDH Activities in the Liver and Stomach

The activities of ADH and ALDH in the liver and stomach were measured. ADH mainly exists in the cytosolic/microsomal fraction, while ALDH exists both in cytosolic/microsomal and mitochondrial fractions. Therefore, cytosolic/microsomal and mitochondrial fractions were prepared from the liver and stomach. First, harvested liver and stomach were immediately homogenized at 4 °C in 100 mM K_2_HPO_4_-KH_2_PO_4_ buffer (pH 7.4) containing 0.25 M sucrose and 0.1 mM EDTA. Then, the homogenate was centrifuged at 600× *g* and 4 °C for 10 min to remove nuclei and cell debris. The supernatant fraction was centrifuged at 10,000× *g* for 15 min, and the supernatant fraction was further centrifuged at 105,000× *g* for 60 min to obtain the cytosolic/microsomal fraction as a supernatant. On the other hand, the pellets were homogenized at 4 °C in 100 mM K_2_HPO_4_-KH_2_PO_4_ buffer (pH 7.4) containing 0.25 M sucrose and 0.1 mM EDTA and centrifuged at 10,000× *g* and 4 °C for 15 min. The precipitates were sonically disrupted in a sonicator at 4 °C for 2 min. The supernatant fraction was then centrifuged at 105,000× *g* for 60 min to obtain the mitochondrial fraction as a supernatant.

The activities of ADH in the cytosolic/microsomal fraction and ALDH in the cytosolic/microsomal and mitochondrial fractions were measured fluorometrically according to the method of Eriksson et al. [19] with some modifications. ADH activity was determined by using an assay mixture (1.2 mL) containing NAD^+^ (1.6 mg) and ALDH (0.32 U) in potassium pyrophosphate buffer (pH 9.0) (F-kit Ethanol, Roche Diagnostics K.K, Tokyo, Japan). After incubating the fraction at 37 °C for 3 min, 100 µL ethanol was added as a substrate of ADH. ALDH activity was determined by using the assay mixture (1.2 mL) containing NAD^+^ (0.32 mg) in potassium pyrophosphate buffer (pH 9.0) (F-kit Acetaldehyde, Roche Diagnostics K.K, Tokyo, Japan). After incubating the fraction at 37 °C for 3 min, 100 µL acetaldehyde was added as a substrate of ALDH.

The initial velocity of NADH production from NAD^+^ was measured (λ_ex_ 340 nm) at 37 °C for 10 min in a microplate spectrometer (CARY 50 UV-Vis spectrometer, VARIAN Inc., Tokyo, Japan). The blank reaction rate without acetaldehyde was also determined. ADH and ALDH activities were normalized to the protein concentration and expressed as the change in nano moles of NADH produced per min per mg of protein.

### 2.11. Statistical Analysis

Statistical analyses were performed using Mac Toukeikaiseki Version 2.0 software package (Esumi, Co., Ltd., Tokyo, Japan). All values are expressed as the means ± SD. Differences among groups were analyzed by the Tukey–Kramer test. Values with significant differences at *p* < 0.05 are expressed with different lower-case letters. A single asterisk indicates a significant difference at *p* < 0.05 compared with 0 min by a Tukey test. Double asterisks indicate a significant difference at *p* < 0.01 compared with 0 min by a Tukey test. The area under the time-concentration curve (AUC) was calculated by the trapezoidal method [20,21].

## 3. Results

### 3.1. Contents of Lentinic Acid in Shiitake-H and Shitake-N

**Figure 5:**
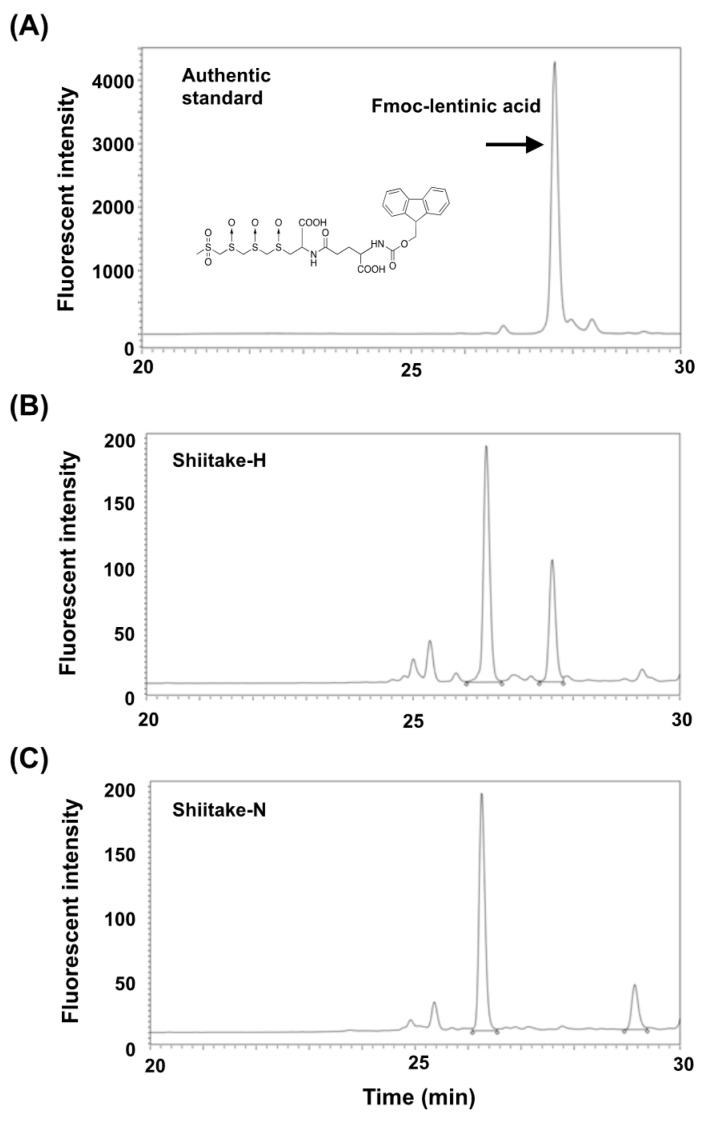
HPLC chromatograms of standard Fmoc-lentinic acid, Fmoc-amino compounds in Shiitake-H and Shiitake-N. (**A**) Standard; (**B**) Shiitake-H; (**C**) Shiitake-N.

Figure 5 shows the HPLC chromatograms of standard Fmoc-lentinic acid, Fmoc-amino compounds in Shiitake-H and Shiitake-N. The lentinic acid content of Shiitake-H and Shiitake-N was 2240 mg (4.6 mmol)/100 g and N.D., respectively.

### 3.2. Changes in Plasma Concentrations of Ethanol, Acetaldehyde, and Acetate after Oral Loading of Ethanol

Table 1 shows the ethanol concentration in the plasma after the oral administration of shiitake extract together with an ethanol solution. The plasma ethanol concentration in all groups showed a peak at 60 min after the administration and then decreased after that. The plasma ethanol concentration in the Shiitake-H group from 30 to 180 min after oral administration was significantly lower than that in the control group at the same time. The AUC for the plasma ethanol of the Shiitake-H group was significantly lower than that of the control group. On the other hand, the plasma ethanol concentration and AUC of the Shiitake-N group showed a lower tendency than that of the control group, though there was no significant difference between them.

Next, we determined the concentration of acetaldehyde in the plasma (Table 2). The plasma acetaldehyde concentration in the control group showed a peak at 120 min after ethanol administration, while that in the Shiitake-H and Shiitake-N groups showed a peak at 60 min after ethanol administration and decreased after that. Furthermore, the plasma acetaldehyde concentration in the Shiitake-H and Shiitake-N groups at 120, 180, and 360 min was significantly lower than that in the control group at the same time after ethanol administration. Furthermore, the AUC for the plasma acetaldehyde of the Shiitake-H and Shiitake-N groups was significantly lower than that of the control group.

As acetaldehyde is further metabolized to nontoxic acetate by ALDH, we next measured the acetate concentration in the plasma (Table 3). The plasma acetate concentration in all groups was significantly increased at 30 min, maintained at an almost constant level up to 180 min, and then that in the Shiitake-H and Shiitake-N groups decreased at 360 min. Furthermore, the plasma acetate concentration in the Shiitake-H and Shiitake-N groups at 360 min was significantly lower than that in the control group at the same time after ethanol administration. However, there was no significant difference at each time point except at 360 min and in the AUC for plasma acetate among all groups.

### 3.3. Changes in Plasma Ethanol Concentration after Injection of Shiitake Extract into the Ligated Loop of the Stomach and Jejunum Together with an Ethanol Solution

To examine the reason for the suppression of elevation in ethanol concentration by Shiitake-H, the effect on ethanol absorption from the stomach and jejunum was measured.

The plasma ethanol concentration in all groups significantly increased at 5 min and gradually increased up to 60 min (Table 4) after its injection into the ligated loop of the stomach. The elevation in plasma ethanol concentration was significantly suppressed in the Shiitake-H group. Furthermore, the AUC for the plasma ethanol of the Shiitake-H group was significantly lower than that of the control group.

The plasma ethanol concentration in the control group showed a peak 5 min after its injection into the ligated loop of the jejunum, and then decreased (Table 5). The plasma ethanol concentration in the Shiitake-H group was significantly lower from 5 min up to 60 min and that in the Shiitake-N group was significantly lower at 5 min than that in the control group. Furthermore, the AUC for the plasma ethanol of the Shiitake-H group was significantly lower than that of the control group.

These results indicate that Shiitake-H suppresses the increase in plasma ethanol concentration by suppressing ethanol absorption in the stomach and jejunum.

### 3.4. Activities of ADH and ALDH in the Liver after Oral Loading of Ethanol

**Figure 6:**
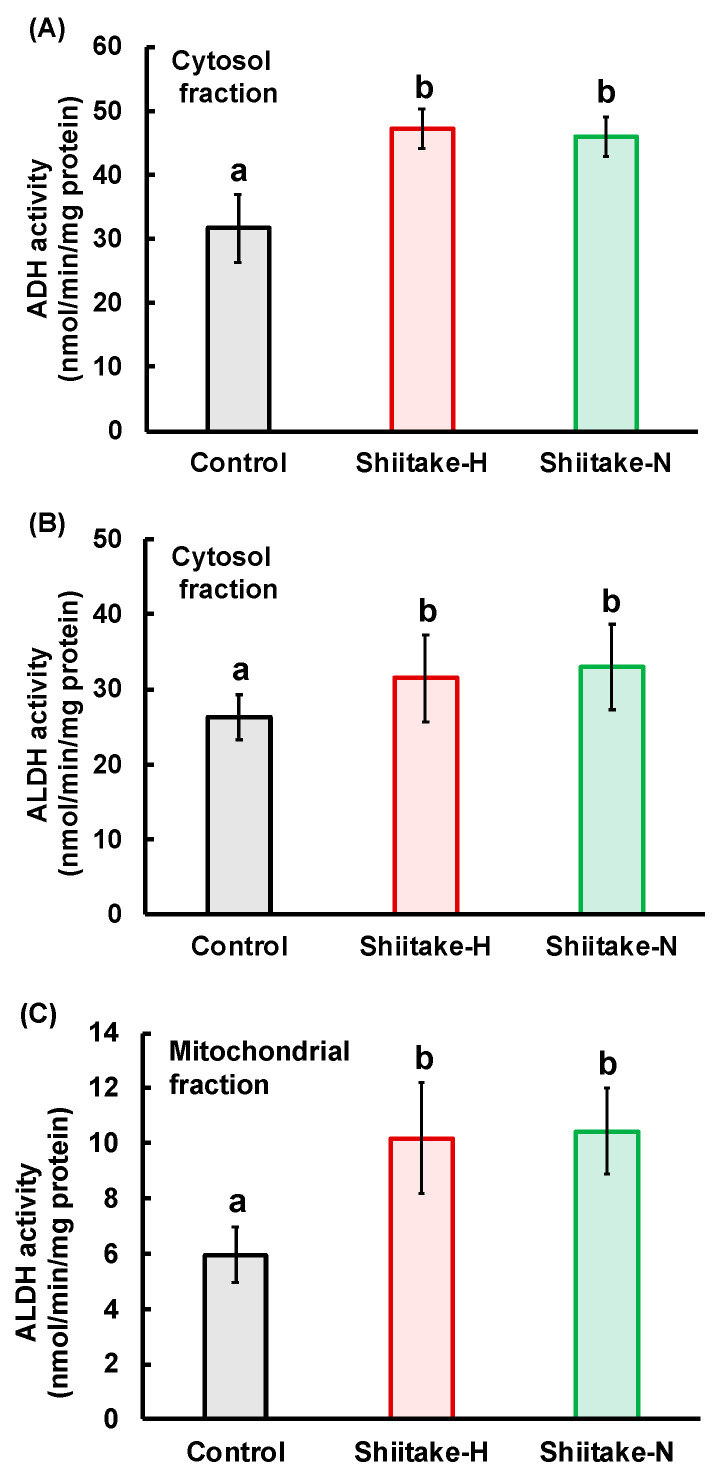
Effect of shiitake extract on ADH and ALDH activities in the liver. (**A**) ADH activity in cytosol fraction (*n* = 4); (**B**) ALDH activity in cytosol fraction (*n* = 4); (**C**) ALDH activity in mitochondrial fraction (*n* = 6). Results with different letters are significantly different at *p* < 0.05. Data are the means ± S.D. ADH: alcohol dehydrogenase, ADLH: aldehyde dehydrogenase.

To examine the reason for the suppression of elevation in ethanol concentration by Shiitake-H, the activities of ADH and ALDH, enzymes that primarily metabolize ethanol were measured. ADH activity in the hepatic cytosolic fraction (Figure 6A), ALDH activity in the hepatic cytosolic fraction (Figure 6B) and ALDH activity in the hepatic mitochondrial fraction (Figure 6C) were significantly higher in the Shiitake-H and Shiitake-N groups than in the control group.

### 3.5. Activities of ADH and ALDH in the Stomach after Oral Loading of Ethanol

**Figure 7:**
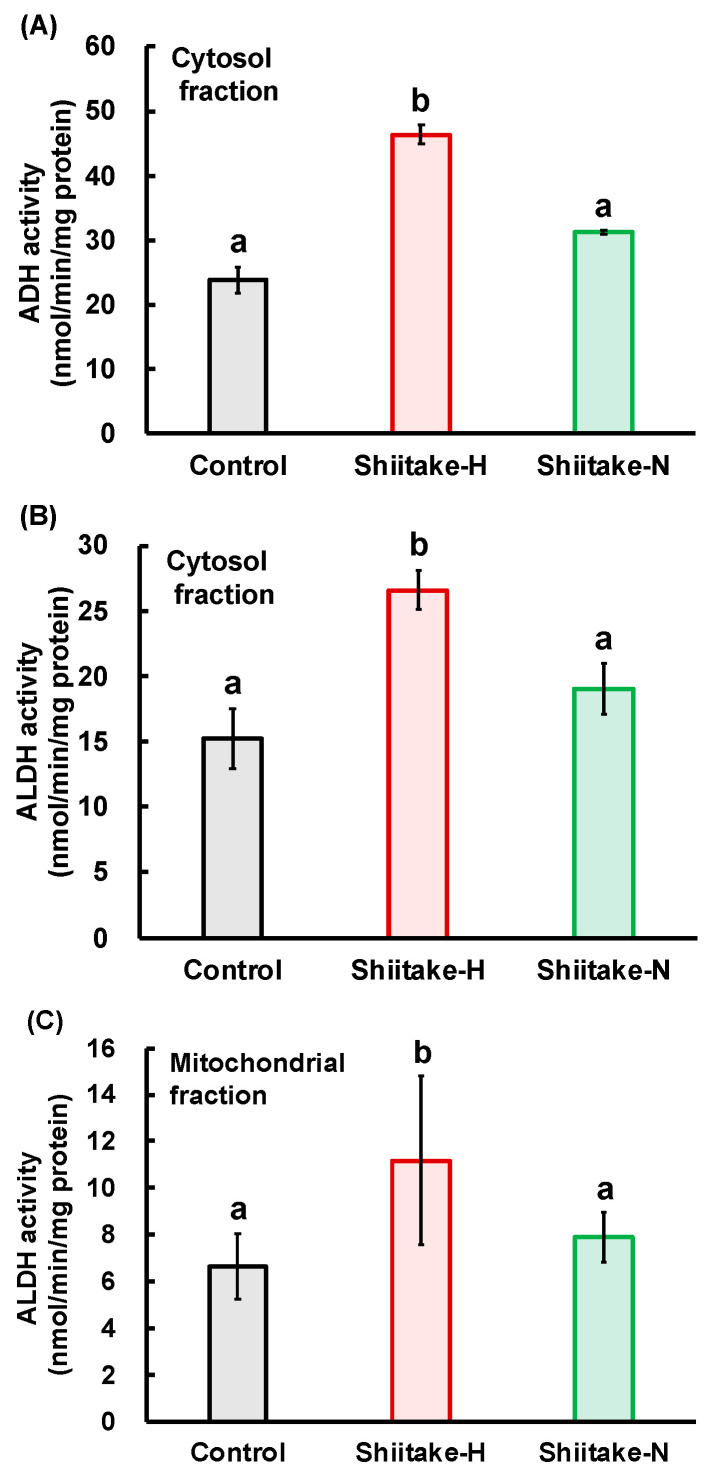
Effect of shiitake extract on ADH and ALDH activities in the stomach. (**A**) ADH activity in cytosol fraction; (**B**) ALDH activity in cytosol fraction; (**C**) ALDH activity in mitochondrial fraction. Results with different letters are significantly different at *p* < 0.05. Data are the means ± S.D.; *n* = 6.

The first pass of ethanol metabolism occurs in the stomach and does not occur during the passage through the liver of absorbed ethanol in the portal blood [22]. Therefore, we evaluated the activities of ADH and ALDH in the stomach after the oral loading of ethanol. ADH activity in the gastric cytosolic fraction (Figure 7A), ALDH activity in the gastric cytosolic fraction (Figure 7B), and ALDH activity in the gastric mitochondrial fraction (Figure 7C) were significantly higher in the Shiitake-H group than in the control and Shiitake-N groups. These results indicate that Shiitake-H accelerates the metabolism of ethanol even in the stomach by increasing ADH and ALDH activities.

### 3.6. Residual Ethanol and Acetaldehyde Concentrations in the Stomach

**Figure 8:**
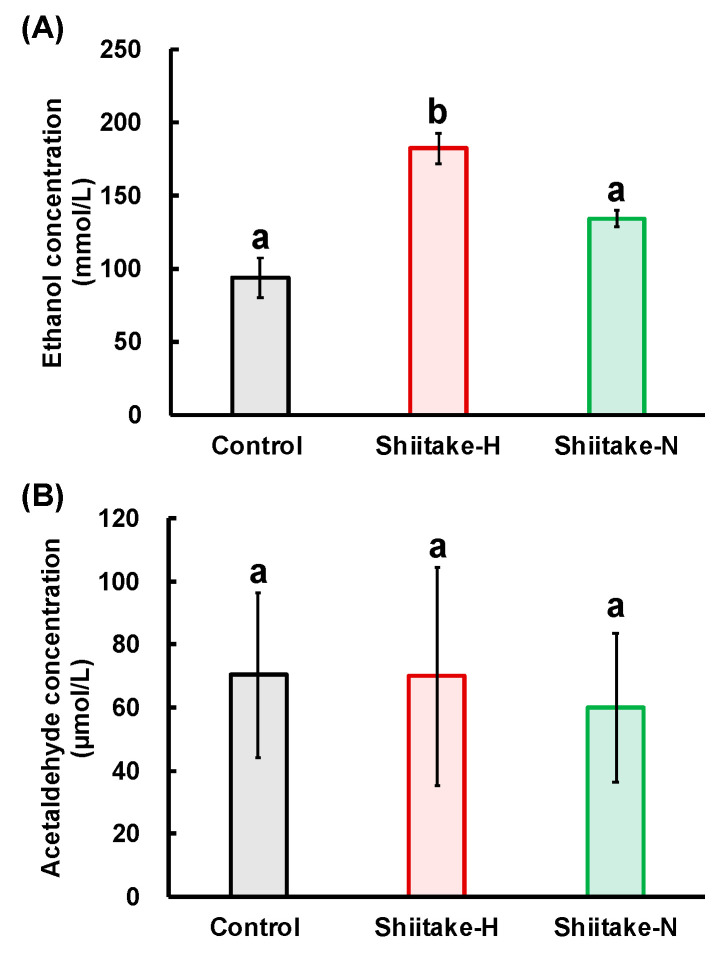
Effect of shiitake extract on residual ethanol and acetaldehyde concentrations in the stomach. (**A**) Ethanol concentration; (**B**) acetaldehyde concentration. Results with different letters are significantly different at *p* < 0.05. Data are the means ± S.D. *n* = 6.

To confirm the prevention of ethanol absorption from the gut by Shiitake-H, residual ethanol and acetaldehyde concentrations in the stomach were measured. Residual ethanol concentration in the stomach was significantly higher in the Shiitake-H group than in the control and Shiitake-N groups (Figure 8A). As ethanol is further metabolized to acetaldehyde by ADH, we next measured the concentration of residual acetaldehyde in the stomach (Figure 8B). However, there was no significant difference in acetaldehyde concentration in the stomach among all groups.

## 4. Discussion

Alcoholic beverages are commonly consumed by adults together with food worldwide, and are enjoyed because alcohol stimulates our appetite and makes meals more delicious. However, excessive intake increases the risk of various diseases. Vegetable and fruit consumption is shown to reduce the risk of alcohol-induced gastric cancer [23] suggesting that food components may affect the absorption and metabolism of ethanol [24]. Therefore, it would be significant to determine food components that prevent alcohol-related diseases. In the present study, we have shown, for the first time, that shiitake extract has a function of suppressing elevation in plasma ethanol concentration. The suppressive effect was higher in the Shiitake-H group than in the Shiitake-N group indicating that lentinic acid, a flavor precursor, would be one of the functional components in shiitake.

The lentinic-acid content in Shiitake-H and Shiitake-N was first determined to ensure that Shiitake-H contained lentinic acid and Shiitake-N did not. This difference in lentinic-acid content in Shiitake-H and Shiitake-N indicates that γ-glutamyl transpeptidase and C-S lyase were inactivated by methanol during the preparation of Shiitake-H, and these enzymes converted lentinic acid to volatile cyclic sulfur compounds during the preparation of Shiitake-N.

The Shiitake-H group being administered shiitake extract containing 300 µmol lentinic acid in 10 mL of 20% ethanol/kg body weight showed lower plasma ethanol elevation from 30 to 180 min after oral administration than the control group (Table 1). We have previously reported that oral administration of a garlic extract with 500 μmol ACSO in 10 mL of 20% ethanol/kg body weight (Garlic-H group) significantly suppressed plasma ethanol elevation 30 to 360 min after oral administration [14]. These results indicate that 300 µmol lentinic acid/kg body weight has a similar function to 500 μmol ACSO/kg body weight.

Two mechanisms can be considered for the suppressive effect of Shiitake-H on elevation of ethanol concentration in the plasma: (1) suppression of ethanol absorption from the gut, (2) promotion of ethanol metabolism in the stomach and liver. We carried out experiments with the aim of determining which of these mechanisms is responsible for this effect of Shiitake-H. As about 10–30% ethanol is absorbed from the stomach and the rest is mainly from the jejunum, we injected ethanol together with Shiitake-H into the ligated loop of the stomach or jejunum, and ethanol concentration in the portal vein was measured. As a result, ethanol concentration in the portal vein was significantly lower in the Shiitake-H group than in the control and Shiitake-N groups from 5 to 60 min after the injection (Table 4 and Table 5). Moreover, as shown in Figure 8A, residual ethanol concentration in the stomach was significantly higher in the Shiitake-H group than in the control and Shiitake-N groups. As no transporters are used for the absorption of ethanol in the gut, the suppressive effect of Shiitake-H on ethanol absorption might be attributable to the retardation of peristaltic movement. Further study is necessary to confirm this assumption. If suppression of ethanol absorption both from the stomach and jejunum is the only factor behind the effect of Shiitake-H, the shape of the curves of acetaldehyde and acetate concentrations would be similar to that of the ethanol concentration with a peak later than 60 min. However, different from acetaldehyde concentration in the control group which shifted the peak to 120 min, that in the Shiitake-H and Shiitake-N groups had a peak at 60 min and decreased sharply after that (Table 2). Previously, we reported that the plasma acetaldehyde concentration in the Garlic-H group showed a peak at 60 min after ethanol administration [14]. Therefore, lentinic acid may have a similar function to ACSO.

On the other hand, the acetate concentration in all the groups was almost the same as that in the control group up to 180 min, and that in the Shiitake-H and Shiitake-N groups decreased sharply at 360 min (Table 3). These results indicate the acceleration of ethanol metabolism in the stomach and liver. Therefore, ADH and ALDH activities were measured.

After ethanol is metabolized to acetaldehyde by ADH, acetaldehyde is metabolized to acetate by two major isoforms of ALDH—ALDH1 in cytosol and ALDH2 in mitochondria. As about half of Asian people do not possess ALDH2 [18], they are more susceptible to the effects of ethanol than Caucasians causing flushing and other vasomotor symptoms after alcohol intake. 6-MSITC in wasabi increases ALDH activity through the induction of mitochondrial ALDH2 expression, but not cytosolic ALDH1A1 in HepG2 cells [16]. In vivo, ACSO in garlic increased activities of mitochondrial ALDH but not cytosolic ALDH in the liver [14]. On the other hand, sulforaphane increased cytosolic and mitochondrial ALDH activities in the liver [15]. Similarly, the activities of cytosolic ADH and cytosolic and mitochondrial ALDH were significantly higher in the Shiitake-H and Shiitake-N groups than in the control group (Figure 6). These results indicate that some components besides lentinic acid in Shiitake-H and Shiitake-N may contribute to the acceleration of ADH and ALDH activities in the liver.

In addition, Shiitake-H also increased the activities of cytosolic ADH and cytosolic and mitochondrial ALDH in the stomach (Figure 7). These findings suggest that the increase in the activities of ALDH would be principally responsible for the fast metabolism of acetaldehyde by lentinic acid in the Shiitake-H.

The residual ethanol concentration in the stomach was higher in the Shiitake-H group than in the control and Shiitake-N groups, but there was no significant difference in acetaldehyde concentrations among all groups. If ADH works on the surface of the stomach, acetaldehyde concentration would increase in the Shiitake-H group. Therefore, this result indicates that ADH would work after ethanol is absorbed from the stomach surface. The differences between Shiitake-H and Shiitake-N in the residual ethanol concentration and the activities of ADH and ALDH in the stomach support the assumption that lentinic acid is a functional component in shiitake that suppress ethanol absorption from the gut. As sulfur-containing compounds such as ACSO, sulforaphane and 6-MSITC are reported to regulate the activities of ALDH [14,15,16], sulfur atoms in lentinic acid may play an important role in exerting the function.

Nuclear factor erythroid 2-related factor (Nrf2) is a transcription factor that up-regulates a diverse array of antioxidant genes. In this regard, Ushida et al. reported that ALDH expression was under the major control of Nrf2, and ALDH activity was increased by the administration of sulforaphane in broccoli and the blood acetaldehyde concentration was decreased [15]. Furthermore, 6-MSITC in wasabi was reported to increase ALDH activity and induce mitochondrial ALDH2 through the Nrf2/ARE pathway in vitro [16]. ACSO also activated the Nrf2/ARE pathway [25] which would increase ADH and ALDH activities. It is, therefore, very likely that lentinic acid affects the Keap1/Nrf2/ARE pathway and accelerates acetaldehyde metabolism in the liver. On the other hand, in the stomach, the activities of cytosolic ADH and cytosolic and mitochondrial ALDH were significantly increased in the Shiitake-H group as compared with the control and Shiitake-N groups (Figure 7). Therefore, the significant suppression of the elevation in ethanol concentration after oral administration of Shiitake-H together with an ethanol solution would be attributed to the acceleration of ethanol metabolism and suppression of ethanol absorption from the gut.

There are several limitations in the present study. First, we did not directly examine if lentinic acid itself affected ethanol absorption and metabolism because of the difficulty of its purification and synthesis. Second, the single oral administration of Shiitake-H and ethanol would represent acute alcoholism. Therefore, further studies should be carried out to examine the preventive effect of Shiitake-H on chronic alcoholism.

## 5. Conclusions

In conclusion, lentinic acid, a shiitake flavor precursor, suppressed elevation in plasma ethanol concentration by preventing ethanol absorption from the gut and accelerating ethanol metabolism in the stomach and liver. In addition to the activities of antioxidant, anti-inflammatory, antihypercholesterolemic, and antithrombotic, shiitake, especially that with a high lentinic acid content, seems to have potential properties to prevent alcohol-related diseases. Furthermore, lentinic acid would be one of the novel functional components besides protein, polysaccharides, dietary fiber, minerals, and vitamins in shiitake for human health.

## Figures and Tables

**Table 1:** Effect of shiitake extract on plasma ethanol concentration after oral administration of ethanol.

	Plasma Ethanol Concentration (mmol/L)		
Time (min)	Control	Shiitake-H	Shiitake-N
0	7.19 ± 2.31 ^a^	6.65 ± 1.83 ^a^	7.07 ± 2.00 ^a^
30	43.41 ± 8.29 ^a,^**	25.53 ± 3.54 ^b,^**	35.79 ± 3.70 ^ab,^**
60	50.48 ± 10.75 ^a,^**	32.54 ± 5.05 ^b,^**	40.90 ± 3.33 ^ab,^**
120	47.28 ± 13.87 ^a,^**	29.78 ± 4.38 ^b,^**	35.95 ± 6.32 ^ab,^**
180	38.01 ± 12.48 ^a,^**	23.06 ± 5.27 ^b,^**	30.09 ± 7.71 ^a,^**
360	10.41 ± 3.93 ^a^	6.71 ± 1.33 ^a^	12.09 ± 5.66 ^a,^**
AUC (mmol·min/L)	9139 ± 2603 ^a^	5089 ± 1042 ^b^	6885 ± 1241 ^ab,^**

Values with different letters at the same time differ significantly at *p* < 0.05 by a Tukey test. Double asterisks indicate a significant difference at *p* < 0.01 compared with 0 min by a Tukey test. Data are the means ± S.D.; all groups (*n* = 6). AUC: the area under the time-concentration curve.

**Table 2:** Effect of shiitake extract on plasma acetaldehyde concentration after oral administration of ethanol.

	Plasma Acetaldehyde Concentration (μmol/L)		
Time (min)	Control	Shiitake-H	Shiitake-N
0	36.64 ± 3.26 ^a^	35.61 ± 1.07 ^a^	36.88 ± 3.67 ^a^
30	49.64 ± 5.73 ^a,^**	44.64 ± 6.95 ^a,^**	46.76 ± 2.31 ^a,^**
60	52.96 ± 3.71 ^a,^**	47.81 ± 2.31 ^a,^**	51.28 ± 4.87 ^a,^**
120	56.87 ± 3.26 ^a,^**	43.07 ± 1.67 ^b,^**	47.46 ± 1.30 ^b,^**
180	51.32 ± 7.29 ^a,^**	38.77 ± 2.03 ^b^	43.57 ± 2.06 ^b,^*
360	38.08 ± 6.75 ^a^	24.94 ± 5.86 ^b,^**	28.98 ± 1.24 ^b,^**
AUC (μmol·min/L)	4100 ± 1660 ^a^	1580 ± 316.2 ^b^	2375 ± 924.1 ^b^

Values with different letters at the same time differ significantly at *p* < 0.05 by a Tukey test. A single asterisk indicates a significant difference at *p* < 0.05 compared with 0 min by a Tukey test. Double asterisks indicate a significant difference at *p* < 0.01 compared with 0 min by a Tukey test. Data are the means ± S.D.; all groups (*n* = 5).

**Table 3:** Effect of shiitake extract on plasma acetate concentration after oral administration of ethanol.

	Plasma Acetate Concentration (μmol/mL)		
Time (min)	Control	Shiitake-H	Shiitake-N
0	0.43 ± 0.06 ^a^	0.40 ± 0.06 ^a^	0.47 ± 0.07 ^a^
30	1.57 ± 0.31 ^a,^**	1.43 ± 0.23 ^a,^**	1.50 ± 0.33 ^a,^**
60	1.70 ± 0.26 ^a,^**	1.60 ± 0.17 ^a,^**	1.59 ± 0.42 ^a,^**
120	1.49 ± 0.48 ^a,^**	1.40 ± 0.38 ^a,^**	1.46 ± 0.40 ^a,^**
180	1.43 ± 0.31 ^a,^**	1.44 ± 0.13 ^a,^**	1.49 ± 0.43 ^a,^**
360	1.35 ± 0.21 ^a,^**	0.76 ± 0.10 ^b^	0.94 ± 0.16 ^b^
AUC (μmol·min/mL)	276 ± 76.6 ^a^	269 ± 49.3 ^a^	262 ± 96.1 ^a^

Values with different letters at the same time differ significantly at *p* < 0.05 by a Tukey test. Double asterisks indicate a significant difference at *p* < 0.01 compared with 0 min by a Tukey test. Data are the means ± S.D.; control and Shiitake-H groups (*n* = 6) and Shiitake-N group (*n* = 8).

**Table 4:** Effects of shiitake extract on plasma ethanol concentration in the stomach after injection of ethanol.

	Plasma Ethanol Concentration (mmol/L)		
Time (min)	Control	Shiitake-H	Shiitake-N
0	11.60 ± 3.56 ^a^	11.53 ± 2.96 ^a^	11.52 ± 3.11 ^a^
5	32.07 ± 5.29 ^a,^*	17.30 ± 3.25 ^b,^*	26.98 ± 3.21 ^a,^*
10	34.32 ± 6.85 ^a,^**	18.54 ± 1.43 ^b,^**	28.49 ± 5.06 ^a,^*
30	37.15 ± 10.02 ^a,^**	19.63 ± 1.43 ^b,^**	33.74 ± 9.02 ^a,^**
60	41.65 ± 10.44 ^a,^**	21.79 ± 2.70 ^b,^**	37.07 ± 12.75 ^a,^**
AUC (mmol·min/L)	1118 ± 502.8 ^a^	359.8 ± 157.9 ^b^	917.3 ± 494.9 ^a^

Values with different letters at the same time differ significantly at *p* < 0.05 by a Tukey test. A single asterisk indicates a significant difference at *p* < 0.05 compared with 0 min by a Tukey test. Double asterisks indicate a significant difference at *p* < 0.01 compared with 0 min by a Tukey test. Data are the means ± S.D.; all groups (*n* = 4).

**Table 5:** Effects of shiitake extract on plasma ethanol concentration in the jejunum after injection of ethanol.

	Plasma Ethanol Concentration (mmol/L)		
Time (min)	Control	Shiitake-H	Shiitake-N
0	8.04 ± 0.39 ^a^	8.12 ± 0.73 ^a^	7.48 ± 1.10 ^a^
5	99.37 ± 15.00 ^a,^**	30.66 ± 8.37 ^c,^**	73.84 ± 5.21 ^b,^**
10	83.75 ± 12.27 ^a,^**	45.24 ± 7.56 ^b,^**	70.44 ± 5.46 ^a,^**
30	57.19 ± 11.17 ^a,^**	27.98 ± 8.02 ^b,^*	53.19 ± 8.73 ^a,^**
60	43.08 ± 8.90 ^a,^**	19.64 ± 3.80 ^b^	36.55 ± 2.30 ^a,^**
AUC (mmol·min/L)	3158 ± 570.4 ^a^	1245 ± 293.2 ^b^	2698 ± 224.8 ^a^

Values with different letters at the same time differ significantly at *p* < 0.05 by a Tukey test. Asterisks indicate a significant difference at *p* < 0.01 compared with 0 min by a Tukey test. Data are the means ± S.D.; all groups (*n* = 4).
